# The Potential of Fermented Water Spinach Meal as a Fish Meal Replacement and the Impacts on Growth Performance, Reproduction, Blood Biochemistry and Gut Morphology of Female Stinging Catfish (*Heteropneustes fossilis*)

**DOI:** 10.3390/life13010176

**Published:** 2023-01-06

**Authors:** Shishir Kumar Nandi, Afrina Yeasmin Suma, Aminur Rashid, Muhammad Anamul Kabir, Khang Wen Goh, Zulhisyam Abdul Kari, Hien Van Doan, Nik Nur Azwanida Zakaria, Martina Irwan Khoo, Lee Seong Wei

**Affiliations:** 1 Department of Aquaculture, Sylhet Agricultural University, Sylhet 3100, Bangladesh; 2 Faculty of Data Science and Information Technology, INTI International University, Nilai 71800, Malaysia; 3 Department of Agricultural Sciences, Faculty of Agro-Based Industry, Universiti Malaysia Kelantan, Jeli Campus, Jeli 17600, Malaysia; 4 Advanced Livestock and Aquaculture Research Group, Faculty of Agro-Based Industry, Universiti Malaysia Kelantan, Jeli Campus, Jeli 17600, Malaysia; 5 Department of Animal and Aquatic Sciences, Faculty of Agriculture, Chiang Mai University, Chiang Mai 50200, Thailand; 6 Science and Technology Research Institute, Chiang Mai University, 239 HuayKeaw Rd., Suthep, Muang, Chiang Mai 50200, Thailand; 7 Department of Agro-Based Industry, Faculty of Agro-Based Industry, Universiti Malaysia Kelantan, Jeli Campus, Jeli 17600, Malaysia; 8 Department of Chemical Pathology, School of Medical Sciences, Universiti Sains Malaysia, Kubang Kerian, Kota Bharu 16150, Malaysia

**Keywords:** fermented water spinach meal, fish meal, female stinging catfish, reproductive performance, health status, sustainable aquaculture

## Abstract

The identification and development of a new plant-based feed ingredient as an alternative protein source to FM have gained the interest of the aquafeed industrial players. Therefore, this study aimed to investigate the physical, biochemical, and bacteriological properties of dietary FWM and the impacts on the growth and reproductive performances of farmed female stinging catfish, *H. fossilis* broodstock. Five experimental diets were formulated with different FWM inclusion (0, 25, 50, 75, and 100%). Fatty acid profiles such as 4:0, 10:0, 20:0, 21:0, 22:0, 24:0, 20:1n9, 18:3n6, 20:3n6, 20:4n6, and 22:6n3 were found in higher levels in FWM compared to the water spinach meal (WM). Meanwhile, there were no significant differences in the physical properties of the FWM experimental diets (*p* > 0.05). Furthermore, the experimental feed with 0%, 25%, 50%, and 75% FWM were more palatable to the broodstock than 100% FWM. The number of total bacteria (TB) and lactic acid bacteria (LAB) in catfish diets exhibited a rising trend with an increase in FWM, while 50% of FWM-fed fish intestines had a significantly (*p* < 0.05) higher TB and LAB than other treatment groups. The growth, feed utilization, and reproductive variables of *H. fossilis* were significantly (*p* < 0.05) influenced by FWM inclusion at various levels. Moreover, the significantly (*p* < 0.05) highest oocytes weight, fertilization, egg ripeness, and ovipositor diameter were observed in the treatment of 50% FWM diet treatment group. In addition, the spawning response was 100% in all treatments except for the control group (66.67%). Significant differences (*p* < 0.05) were found in the hematological and serum biochemical indices in most treatment groups. In addition, the histological analysis of *H. fossilis* midintestinal tissue indicated that the fish fed with a 50% FWM diet had an unbroken epithelial barrier with more goblet cell arrangements and a well-organized villi structure and tunica muscularis compared to other treatment groups. These outcomes suggested that FWM at 50% inclusion is an adequate protein supplement for fish feed, resulting in better growth, reproductive performance, and health of *H. fossilis* broodstock development.

## 1. Introduction

Stinging catfish (*Heteropneustes fossilis*) is a valuable and commercially important aquaculture species in Bangladesh and many parts of Southeast Asia. This species has recently gained substantial interest from the hatchery industry due to its high growth rate, survivability, and nutritional value [1]. Stinging catfish is an omnivorous fish that requires a moderate protein level in its diet for optimal reproductive output and growth. Additionally, studies have reported that an average of 54% of a stinging catfish population are used as broodstock for spawning [2,3,4] and maintained three to four times to achieve seed production goals in hatcheries. Nevertheless, poor reproductive performance, lack of quality feed, and husbandry requirements are major issues in managing the female stinging catfish broodstock culture. Therefore, aquaculture nutritionists consistently search for novel, high-quality alternatives of plant and animal origin [5,6,7,8,9,10,11,12,13,14,15]. Moreover, [16,17,18] it has been reported manipulating the nutrient composition of broodfish diets improves spawning response, lowers the number of broodfish required to achieve production goals, and enhances egg production and quality. 

Broodstock and its egg quality are critical in ensuring superior fry production. Several measures could be taken by aquaculturists to produce the finest broodstock, such as providing the fish with good quality and protein-rich diet. Currently, fish meal (FM) is considered the best animal-derived fish feed ingredient and protein source for broodstock reproductive development. Nevertheless, insufficient supply, unavailability, high price, and low quality can result in the partial or complete replacement of FM with plant-derived ingredients [19]. Furthermore, plant proteins are the best FM alternative because of the abundance and affordable price [20]. Thus, this study aimed to explore and develop a low-cost and sustainable protein source from water spinach meal (WM) as a FM replacement. Water spinach (*Ipomoea aquatica*) is a semiaquatic tropical plant found in most water bodies, particularly within the haor basin of Bangladesh and most Asian countries. This aquatic weed is highly nutritious [21]. For instance, the aquatic weed meal (AWM) contains 26.5 to 32.5% crude protein and is rich in amino acids, minerals, and vitamins [22]. Therefore, aquatic weeds are valuable protein sources and fish feed ingredients for the aquafeed industry [2,22].

Studies on aquatic weeds, such as water spinach, as a viable protein source for fish feed formulation, remain scarce. Furthermore, plant-based ingredients contain significant amounts of anti-nutritional factors (ANFs); hence, it is essential to practice caution when incorporating this raw material in fish feed to prevent adverse effects on fish growth and health [23,24]. One of the most effective ways to eliminate ANFs is semisolidify fermentation (SSF), which could also improve the nutritional profile of plant ingredients [12,13,25]. The SSF of water spinach is a unique method of delivering beneficial microbes into the fish intestine, which significantly impacts fish growth performance and feed conversion rate [26,27]. Several studies have investigated the impacts of water spinach meal (WSM) at various inclusion levels on the growth performance of fish [28,29,30], but none have identified the effects of this plant-based protein on fish reproductive performance. Specifically, data on the utilization of fermented water spinach meal (FWM) as a potential feed ingredient for the reproductive performance of female stinging catfish broodstock in the aquaculture sector remains lacking. Therefore, the current research attempted to address this issue by investigating the impacts of FWM inclusion at varying proportions in diets on the growth, reproductive performance, and health status of *H. fossilis* broodstock.

## 2. Materials and Methods

### 2.1. Broodfish Collection and Holding

A group of sexually mature *H. fossilis* (average weight: 64.00 ± 0.50 g) was purchased from a commercial fish farm and immediately transported to the Aquaculture Laboratory, Faculty of Fisheries, Sylhet Agricultural University. The broodfish used in this study were of standard size and similar to the ones used by farmers and other researchers in Bangladesh. Initially, the fish were acclimatized and maintained in a fiberglass tank (2000 L) and provided with a basal diet once daily (30% crude protein, 6% crude lipid) at 1% of body weight. Two weeks before the feeding trial, 150 fish were individually weighed and distributed randomly into 15 circular plastic tanks (100 L/tank) with a stocking density of 10 fish/tank and a sex ratio of 4 females: 1 male. In addition, a bottom cleaning system was employed for waste removal from the tanks. The experiment consisted of five treatments and was conducted in triplicates.

### 2.2. Preparation of Fermented Water Spinach Meal (FWM)

The raw water spinach was collected from the haor basin of Sylhet and sun-dried until the moisture content was <10%. The dried samples were ground and fermented according to Zulhisyam et al. [12,13,25] with some modifications. Subsequently, the WM fine powder was mixed with 0.001% of *Lactobacillus acidophilus* (Sigma, St. Louis, MO, USA) and 10% molasses. The mixture was placed in a plastic container for 21 days to complete the SSF process in a specific quantity.

### 2.3. Experimental Diets and Feeding Trial

Five experimental diets (30% crude protein) were prepared conventionally with different FWM inclusion levels [0% (control), 25%, 50%, 75%, 100%]. The FWM powder was finely grounded and mixed with other ingredients: FM, soybean meal, corn germ meal, distillery dry grain soluble, rice polish, wheat bran, palm oil, soybean oil, vitamin and mineral premix, and carboxymethyl cellulose (binder). The mixture was then pelleted (floating) via an extruder (LM40-floating fish feed machine, Henan Lima Machinery Manufacture Co. Ltd., Zhengzhou, China) to produce feed with a diameter of 2 mm diameter. Subsequently, the pellets were oven-dried at 60°C, cooled at room temperature, packed in airtight plastic bags, and stored at −15 °C until use. The broodstock was fed at 2% body weight twice daily (9.00 am and 5.00 pm) with the respective diets for 90 days prior to the spawning. The feed formulation and proximate composition [31] of the experimental diets are shown in Table 1.

### 2.4. Fatty Acid Profile Analysis

The fatty acid profiles of WM and FWM were determined using LCMS-MS following the standard protocol by Kabir et al. [18]. The experiment was conducted in triplicates. 

### 2.5. Recording of Tank Water Hydro-Ecological Variables

The temperature, salinity, pH, water pressure, total dissolved solids (TDS), conductivity, and dissolved oxygen of the broodstock holding tanks water were recorded weekly for 90 days using a multiparameter probe (HI 9828, YSI Incorporation, Yellow Spring, OH, USA). Meanwhile, the ammonia, nitrite, and nitrate levels were monitored using a Hach kit (HI 28049, HACH, USA).

### 2.6. Measurement of Physiological Properties of Experimental Diets

The physical parameters and palatability examination of experimental fish diets were estimated according to the standard method by Zulhisyam et al. [25] with some modifications:

i. Expansion ratio (%) = 100 × (Average diameter of FWM treated feed particle—Average diameter of original commercial feed)/Average diameter of original commercial feed
ii. Bulk density (kgm^−3^) = Weight of feed in a cylinder/Total volume occupied
iii. Pellet durability index (%) = 100 × (Weight of the residual feed pellets on the sieve/Initial weight of pellets before tumbling)
iv. Water stability (%) = 100 × (Weight of retained whole feed pellets after immersion/Initial weight of feed pellets)
v. Floatability (%) = 100 × (Average numbers of floating feed after the test period/Average numbers of initial feed)

### 2.7. Growth and Reproductive Variables Calculation

At the end of the experiment, five fish from each group were randomly selected for evaluation. The genital papillae of each female were observed, and the diameter was measured. At the end of the feeding trial, the final weight and survival of the broodfish were recorded. The fish were then euthanized and dissected to collect and weigh the following tissue samples: fat, ovary, viscera, and liver. The oocyte amount, weight and diameter, ovary weight, relative fecundity, fertilization, and ripe oocyte rate were estimated as per Kabir et al. [10,12]. Additionally, the following parameters were calculated to determine the broodstock growth and reproductive performance:

i. Broodfish survival (%) = 100 × (Number of broods that survived)/(Total numbers of broods at the start of the experiment)
ii. Weight gain, WG (g) = 100 × (Final weight–Initial weight)/(Initial weight)
iii. Specific growth rate, SGR (%/day) = 100 × ln (final weight)^−ln^ (Initial weight)/(Days of the experiment)
iv. Feed conversion ratio, FCR = (Total feed consumed)/(Live weight gain)
v. Feed conversion efficiency, FCE = (Live weight gain)/(Total feed consumed)
vi. Protein efficiency ratio, PER = (Live weight gain)/(Crude protein fed)
vii. Gonadosomatic index, GSI = 100 × (Gonad weight)/(Body weight)
viii. Hepatosomatic index, HSI = 100 × (Liver weight)/(Body weight)
ix. Visceral somatic index, VSI = 100 × (Viscera weight)/(Body weight)
x. Intraperitoneal fat, IPF = 100 × (Fat weight)/(Body weight)
xi. Spawning response (%) = 100 × (Number of hormone (Ovupin; 0.5 mL/kg fish)-injected fish spawned)/(Total number of hormone-injected female)
xii. Ovulation time (h) = Total time until ovulation–Time at first injection
xiii. Relative fecundity (eggs. kg^−1^ female) = (Total number of eggs in the female ovary)/(Female weight)
xiv. Egg fertilization rate (%) = 100 × (Numbers of fertilized eggs in subsample)/(Total number of eggs in subsample)
xv. Ripe egg (%) = 100 × (Number of eggs with yolk position near one edge of the egg)/(Total number of eggs counted)

### 2.8. Bacterial Load Estimation in Diets and Fish Intestine

Total bacteria (TB) and lactic acid bacteria (LAB) in the collected samples were determined following the method of Zulhisyam et al. [25] and Kari et al. [13] with some modifications. The sample was weighed (±1 g) and vortexed with a 9 ml sterile solution. The mixture was then serially diluted to 10^−10,^ and 100µl of the suspended solution was spread onto tryptic soy agar (TSA) (HiMedia, Maharashtra, India) for TB count and de Man, Rogosa, and Sharp (MRS) HiMedia, India) agar to determine the LAB load. Finally, the plates were incubated at 37 °C for 48 h, and the visible colonies were enumerated.

### 2.9. Blood Hematology and Biochemistry Assays

A total of three broodfish from each treatment group were randomly sampled and anesthetized with tricaine methanesulfonate (MS_222_) at the end of the feeding trial. Blood was collected from the caudal vein of every catfish using a 1 ml sterile syringe and stored in heparinized EDTA K_3_ tubes. Subsequently, the blood samples were assessed for hematological indices via the automated hematology analyzer (Mythic 18 Vet, USA). In addition, the serum was separated by centrifuging the blood samples at 3000 rpm for 10 min to determine the biochemical parameters, such as glucose (GLU), creatine (CREA), total bilirubin (TBIL), serum glutamic pyruvic transaminase (SGPT), serum glutamic oxaloacetic transaminase (SGOT), albumin (ALB), alkaline phosphatase (ALKP), cholesterol (CHOL), total protein (TP), globulin (GLOB), alanine aminotransferase (ALT), aspartate aminotransferase (AST), and gamma-glutamyl transferase (GGT) by employing an automatic blood analyzer (Beckman Coulter AU680, USA).

### 2.10. Histological Examination of the Midgut

A total of three fish from each treatment group were randomly selected and deprived of feeding for 72 h for gut emptying. The fish were then euthanized and dissected to collect their gut tissue samples and fixed in 10% buffered formalin solution. Each sample was cut into 1 mm transverse sections in triplicates, processed and dehydrated through a series of ethanol, cleaned in xylene, embedded in paraffin, sectioned (5–8 μm), mounted, and stained in routine hematoxylin and eosin (H&E). Each tissue slide was examined under a light microscope (Leica DMIL-LED, Germany). Finally, the images of each section from the fish were digitized using an imaging software (Cellsens, Netherlands).

### 2.11. Statistical Analysis

All the collected data were tested for normality before further analysis. First, Levene’s test was used to examine the variance homogeneity to confirm the data normality and homogeneity. Subsequently, the one-way analysis of variance (ANOVA) was performed using the Statistical Package for the Social Sciences (SPSS) version 20.1 (IBM, USA) to evaluate the differences between control and treatment groups for all study parameters. If variance homogeneity was met, Duncan’s test was performed on the data. If not, the data was examined using Tamhane’s T2 test. Finally, the results were presented as mean ± SD at a significant level of *p* < 0.05.

## 3. Results

### 3.1. Fatty Acid Profile of Test Ingredients

The fatty acid profile of WM and FWM is shown in Table 2. Fatty acids such as 8:0, 16:0, 18:0, and 18:1n9 were present in both feed ingredients. However, 4:0, 10:0, 20:0, 21:0, 22:0, 24:0, 20:1n9, 18:3n6, 20:3n6, 20:4n6, and 22:6n3 fatty acids were mostly found in FWM compared to WM. Furthermore, 16:0 fatty acid was the highest in both feed ingredients in this study.

### 3.2. Physical Properties and Palatability Examination of Catfish Diets

The physical characteristics and palatability examination of experimental diets are illustrated in Table 3. There were no significant differences in the physical properties of FWM experimental diets in feed dimension, expansion rate, bulk density, pellet durability index, floatability, and water stability (*p* > 0.05. Nevertheless, the palatability evaluation indicated that the experimental diets with 0, 25, 50, and 75% FWM were more palatable to broodfish, while the palatability substantially reduced 100% FWM inclusion.

### 3.3. Quantification of Bacterial Loads in Catfish Diets and Intestine

The TB and LAB of FWM-based experimental diets and broodstock intestines are presented in Table 4. The TB and LAB counts in broodstock diets and intestines were significantly different (*p* < 0.05) between the FWM diet groups. Furthermore, the lowest TB and LAB counts were observed in the control feed. Meanwhile, the gut microbial loads were the highest in the broodstock group fed with 50% FWM diets. Nonetheless, no significant differences (*p* > 0.05) were found in the intestinal TB and LAB quantity in 75% and 100% FWM treatment groups.

### 3.4. Hydrological Variables in the Broodstock Holding Tank Water 

The water parameters in the respective tanks are presented in Table 5. Most hydrological parameters, including temperature, salinity, dissolved oxygen, pH, nitrite, and nitrate, were not significantly different between FWM groups (*p* > 0.05). Conversely, water pressure, conductivity, TDS, and ammonia levels were significantly different between the treatment groups (*p* < 0.05), but no specific trend was observed throughout the experiment.

### 3.5. Growth and Reproductive Performance and Egg Quality Assessment 

Table 6 demonstrates the growth and reproductive performance of stinging catfish broodstock fed with diets containing various proportions of FWM. In this study, the female broodfish growth and reproductive parameters (weight gain, SGR, fecundity, GSI, HSI, VSI, IPF, spawning response, ovulation time, and ovipositor diameter) were significantly (*p* < 0.05) influenced by the different percentages of FWM in the experimental diets. Additionally, the parameters of final weight gain, SGR, fecundity, and GSI of female broodfish were significantly (*p* < 0.05) increased with FWM levels in the experimental diets. Despite that, the mean IPF value and ovulation time were significantly (*p* < 0.05) higher in the control broodfish than in other treatment groups. Furthermore, there were no significant changes in broodfish survivability (*p* > 0.05) between the groups. Meanwhile, the feed utilization indices, such as FCR, FCE, and PER, were significantly (*p* < 0.05) improved with FWM inclusion in the diets. The lowest mean value for spawning response (66.67%), GSI (9.99%), and fecundity (11.82 × 10^4^) were observed in fish that fed on a 0% FWM diet. Conversely, the significantly (*p* < 0.05) highest HSI, VSI, and OD were recorded by the group fed with a 50% FWM diet. Furthermore, the egg quality parameters, such as fertilization, egg weight, and ripeness, were significantly different between the treatment groups. The highest oocyte weight, fertilization, and ripeness were recorded by the broodfish fed a 50% FWM diet, and no adverse effects were observed on oocyte weight and ripeness in groups supplemented with 50% and 75% FWM diets.

### 3.6. Hematological Analysis

Table 7 presents the hematological parameters of broodstock fed with diets supplemented with FWM at different levels. The broodstock that was fed with 50% FWM had significantly (*p* < 0.05) higher white blood cell (WBC), lymphocytes (LYM), monocytes (MON), and eosinophil (EOS) than other FWM treatment groups. Nevertheless, the mean values of basophil (BAS), hematocrit (HCT), and procalcitonin (PCT) were not influenced by different levels of FWM. Furthermore, granulocytes (GRA) were the highest in fish fed with a 100% FWM diet. The significantly (*p* < 0.05) lowest red blood cell (RBC) and hemoglobin (HGB) were exhibited by the control group (0% FWM diet). Meanwhile, the mean corpuscular volume (MCV), mean corpuscular hemoglobin (MCH), and mean corpuscular hemoglobin concentration (MCHC) were statistically different between the treatments, with the highest levels recorded by the 50% FWM diet group. Moreover, the mean platelet volume (MPV) was significantly (*p* < 0.05) altered but did not demonstrate an increasing or decreasing trend. In addition, a significantly (*p* < 0.05) lower red cell distribution width (RDW) was observed in the broodstock fed with 100% FWM compared to other groups. The control fish had significantly (*p* < 0.05) lower platelet (PLT) and platelet distribution width (PDW) than other diet groups.

### 3.7. Plasma Biochemical Assays

Serum biochemical parameters of the broodstock are presented in Table 8. The mean glucose, creatinine, total bilirubin, albumin, alkaline phosphatase, total protein, globulin, alanine aminotransferase, aspartate aminotransferase, and gamma-glutamyl transferase significantly (*p* < 0.05) increased with FWM levels in diets. Moreover, the significant (*p* < 0.05) and highest serum glutamic pyruvic transaminase and serum glutamic oxaloacetic transaminase were observed in broodstock fed with a 50% FWM diet. However, the cholesterol levels varied among the FWM treatment groups without any trend.

### 3.8. Histomorphology of Mid-Intestine Tissue

**Figure 1:**
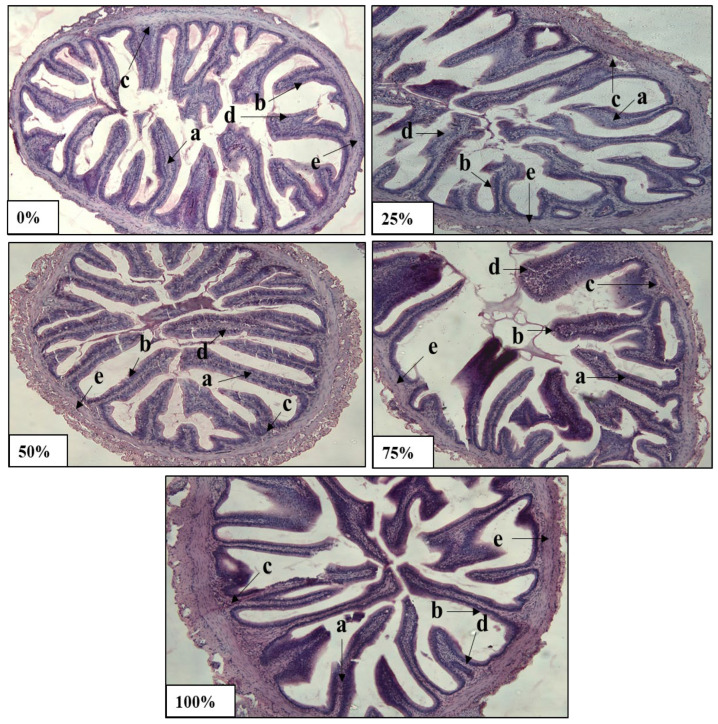
The histological images of the midintestine of stinging catfish broodstock fed with diets with FWM at different inclusion levels (0%, 25%, 50%, 75%, and 100%). The histological alterations were identified in the (a) lamina propria, (b) lamina epithelial mucosae, (c) stratum compactum, (d) goblet cells, and (e) tunica muscularis. Magnification: 10×. Scale bar: 200 μm.

Figure 1 demonstrates the histological observation of the mid intestine of fish fed different levels of FWM experimental diets. The histopathological assessment, including the lamina propria, lamina epithelial mucosae, stratum compactum, goblet cell, and tunica muscularis, revealed that the fish fed with a 50% FWM diet had an unbroken epithelial barrier with more goblet cell arrangements and improved villi structure, tunica muscularis compared to other treatment groups. Nevertheless, regular distribution of lamina epithelial mucosae, tunica muscularis, and goblet cells were observed in all the experimental groups. 

## 4. Discussion

To the best of our knowledge, this is the first study that evaluated the impacts of replacing FM with FWM on the reproductive performance, egg quality, and health status of female stinging catfish broodstock development. Moreover, the FWM is a novel and promising protein source; thus, identifying the most effective level of inclusion in fish feed could reduce the dependence on FM, eliminate ANFs, and boost economic growth. Therefore, this study investigated the fatty acid profile and physical, biochemical, and bacteriological studies of FWM diets. In addition, the hematological and biochemical assays and gut morphology of broods **of** stinging catfish were assessed to elucidate the potential of FWM as a FM replacement. 

The literature [18,32] has reported that dietary lipid and fatty acid molecules influence fish fecundity, offspring viability, fertilization rate, and the percentage of deformed larvae. The study findings indicated that FWM is richer in most fatty acid groups than WM. Resultantly, FWM inclusion at different percentages in the experimental diets might increase the fatty acids deposition into the liver, which could be useful as energy sources for vitellogenin production and reproduction in the fish and ensure their egg quality. The dietary supplementation of plant fatty acid in earlier studies has been reported in different fish species such as *Acanthopagrus latus* [33], *Channa striatus* [17], *Danio rerio* [34], and *Pangasianodon hypophthalmus* [18].

Good physiological characteristics that prevent excessive dust and debris production are crucial for pellets to withstand handling, transportation, and pneumatic conveyance [35]. In the commercial feed industry, pellets with excellent physical properties are a prerequisite for aqua-feed production. In this study, the physical properties of different FWM experimental diets did not significantly vary from each other, which could be due to the identical pellet size. Previous reports have stated that the expansion ratio, bulk density, and water stability of the pellet increased with size [25,35,36]. In contrast, PDI and floating rate exhibited a decreasing trend with increasing feed diameter [25,35,37]. Additionally, the present findings suggested that palatability was consistent among the different diets containing 0, 25, 50, and 75% FWM but decreased drastically when 100% FWM was included in the fish feed, which aligned with previous studies [25,38]. The lower palatability for the 100% FWM diet could be caused by the greater abundance of indigestible compounds and ANFs. Furthermore, Gamboa-Delgado & Marquez-Reyes [39] stated that the dietary inclusion of beneficial bacteria substantially increased feed palatability. Yamamoto et al. [40] also suggested that the palatability characteristics of diets were dramatically reduced due to ANFs, high crude fiber, and other toxic materials.

The bacteriological findings indicated that the TB and LAB levels in the experimental diets were significantly enhanced with increasing levels of FWM (0 to 100%), which was previously reported in a study on African catfish, *Clarias gariepinus* diets [25]. Furthermore, the significantly highest TB and LAB were recorded in 50% FWM-fed broodfish intestines compared to other treatment groups, consistent with the findings by Zulhisyam et al. [25]. The present study introduced probiotic bacteria into the fish gut via feed, resulting in outstanding broodfish growth, reproductive performance, and health. The delivery of lactic acid bacteria into the fish intestine has increased growth [12,13,41,42,43,44,45] and reproductive status [42,43,44] in different fish. For instance, Aliyu et al. [45] revealed that fermenting plant ingredients significantly eliminated disease-causing bacteria from fish intestines. Therefore, higher levels of beneficial bacteria in the intestine improve growth, reproductive performance, survival, and health status among broodstock.

The water parameters in the broodstock holding tank did not differ significantly in temperature, salinity, DO, pH, nitrite, and nitrate, except for water pressure, conductivity, TDS, and ammonia. Utilizing water from the same source help to ensure consistency in water quality parameters, but minor changes may be present due to feeding different FWM diets to the broodstock. Furthermore, fish production, reproduction, and feeding are highly affected by water quality [46,47]. Over the study period, the hydrological variables were within the recommended levels for stinging catfish growth and reproduction, as suggested by Bhatnagar et al. [48], Bhatnagar & Singh [49], and Ekubo & Abowei [50]. Ideally, optimal water parameters would reduce stress, improving broodstock survival rate.

Based on the statistical analysis in this study, the development of maternal feed from FWM with 0.001% *Lactobacillus* improved the nutritional profiles of fish diets, which is crucial for optimal growth, reproductive performance, and egg quality. Likewise, Adejuwon et al. [51] reported that the fermentation of plant-based ingredients enhanced the protein content of feedstuffs. In the present study, the protein percentage of experimental diets was approximately 30%, which fulfilled the protein requirement of stinging catfish broodstock. In addition, the FWM-fed fish groups recorded the highest weight gain, survival rate, and SGR compared to the control group, consistent with earlier studies [12,13,25,52,53]. The positive outcome may be attributed to the high-quality protein in FWM. 

Fecundity estimates the number of oocytes produced per kg of female broodstock at one time. The catfish fecundity increased with FWM levels, with estimated mean values of 1.5 to 3 folds higher than the control group. Muyinda et al. [54] reported similar outcomes when *C. gariepinus* was fed soybean meal-based diets. Likewise, *Hemibagrusnemurus* [55] and *Pangasianodon hypophthalmus* [16] demonstrated similar results when fed with plant protein diets. Moreover, the GSI mean values were significantly elevated with FWM inclusion at different levels in fish feed, which aligned with a study by Kabir et al. [18]. Moreover, significantly highest HSI and VSI values were recorded in the group fed with 50% FWM diet. Similarly, Kari et al. [12] supported these findings by demonstrating that the fish fed with 50% fermented soy pulp diet had better HSI and VSI than other treatment groups. In this study, the fish provided with 50, 75, and 100% FWM recorded relatively lower IPF values than the other treatments, indicating that the fat deposition in the liver was greatly reduced and actively utilized by fish for their gonadal development. 

The current study also investigated how the dietary inclusions of FWM at varying degrees influenced fish feed utilization. The feed conversion rate was higher in FWM treatment groups, indicating that this ingredient may contain more digestible compounds that are readily absorbed in the fish intestine, as reported by Yanto et al. [53], Yousif et al. [28], and Kari et al. [13]. In this study, spawning response and fertilization rates were up to 100% and 98% for all treatment groups, except for control, which paralleled previous reports [16,18,56,57]. Furthermore, the broodfish ovipositor diameter, oocyte weight, and egg ripeness varied highly among the FWM-treated diets. Nonetheless, fish fed with 50% FWM diet exhibited the highest mean value. It was also concluded that the dietary inclusion of protein at various percentages in the experimental feed substantially impacted the ovipositor diameter [16,58], egg weight, and ripeness [16]. These results suggest that FWM as a protein supplement in stinging catfish diets may improve their growth, reproduction, and egg quality.

Broodstock that fed on 50% FWM had significantly higher WBC and RBC levels than other treatment groups, whereas HCT values were not affected by different levels of FWM inclusion in fish feed. These results are similar to a previous report [13]. On the contrary, Ismail et al. [59] observed no variations in these parameters when different levels of fermented *Azolla* meal were fed to Nile tilapia. Meanwhile, Ozovehe [60] claimed that WBC, RBC, and HCT are indicators of antinutritional toxicity and health condition. Stinging catfish that fed on 50% FWM demonstrated high levels of WBC, thus indicating a potentially improved defence mechanism. Furthermore, FWM at different levels of dietary inclusion groups significantly impacted broodfish hemoglobin levels, which were also observed in *Huso huso* [61,62] and *Clarias gariepinus* [13]. Likewise, there were significant variations in GRA, MCV, MCH, MCHC, MPV, RDW, PLT, and PDW levels among African catfish fed with graded levels of fermented soy pulp [13]. Despite that, other plant-based ingredients fed to fish did not exhibit any discernible variation in MCV, MCH, and MCHC mean values [61,62].

Glucose, creatinine, and total bilirubin levels significantly increased with FWM supplementation, while urea in plasma was the lowest in stinging catfish broodstock fed with 50% FWM diet. Similar findings were reported in African catfish *C. gariepinus* fed with *Lactobacillus* fermented soy pulp [13]. Furthermore, serum glutamic pyruvic transaminase and glutamic oxaloacetic transaminase levels were statistically different without any specific trend. Meanwhile, the broodstock group fed with 100% FWM displayed the highest albumin, alkaline phosphatase, total protein, and globulin levels, consistent with a previous study on African catfish [13]. In contrast, Magouz et al. [63] reported no significant variations in the mean of ALB, TP, and GLOB when various quantities of *Azolla* meal were administered in *O. niloticus* diets. Besides serving as plasma transporters, ALB and GLOB guarantee a healthy system [64].

In this study, the fish cholesterol level was greatly influenced by the various proportions of FWM-treated diets, which corresponded with the study by Nguyen et al. [65]. In addition, alanine aminotransferase (ALT) and aspartate aminotransferase (AST) are important indicators of stress [66]. In the present study, ALT, AST, and gamma-glutamyl transferase levels were substantially different among the broodfish groups, consistent with the findings by Kari et al. [13]. In contrast, Ismail et al. [59] reported no differences in ALT and AST levels in a study on *O. niloticus*. In summary, the changes in biochemical indices reflect variation in broodstock health in different treatment groups.

The omnivorous fish gut is sensitive to feed and has a greater surface area for nutrient absorption attributed to structures such as the villi, microvilli and goblet cells arrangement [67,68]. Shiu et al. [69] reported that histomorphological examination of the fish gut provides insight into its nutritional status. Based on the current findings, the intestinal structure of broodstock fed with 50% FWM diet had an unbroken epithelial barrier with more goblet cells and well-arranged villi structure and tunica muscularis compared to other groups. Likewise, these outcomes were observed in African catfish fed with 50% fermented soy pulp diet [13]. Furthermore, scientists reported that dietary supplementation of fermented plant-based ingredients in diets ameliorated the fish gut morphology [70,71,72,73,74]. Another study documented that highly abundant microvilli and villous folding increase the surface area of the gastrointestinal tract, thus, facilitating nutrient absorption [75]. Nonetheless, the broodfish fed with 75 and 100% FWM in this study were adversely impacted in terms of gut health, which could be attributed to high levels of ANFs. Meanwhile, the gut of fish fed with 50% FWM diet displayed long villi structure and small lumen space, indicating improved gastrointestinal health and nutrient absorption capacity.

## 5. Conclusions

Based on the reproductive parameters and health status of female broodstock in this study, it was concluded that 50% FWM dietary inclusion enhanced the reproductive performance of farmed stinging catfish. This research provided insights into using novel plant-based proteins, such as water spinach, as a low-cost feed ingredient for healthy fish feed production. In addition, the study findings will aid future researchers in critical areas, such as FWM delivery via pellets for freshwater fish.

## Figures and Tables

**Table 1:** Feed formulation and proximate composition (dry matter basis) of the experimental diets.

Ingredients (g/kg)			Diets (%)		
	0	25	50	75	100
LFM ^1^	380	285	190	95	0
FWM ^2^	0	95	190	285	380
Soybean meal	250	250	250	250	250
CGM ^3^	45	45	45	45	45
DDGS ^4^	35	35	35	35	35
Rice polish	25	25	25	25	25
Wheat bran	155	155	155	155	155
Palm oil	20	20	20	20	20
Soybean oil	30	30	30	30	30
Vitamin and mineral premix ^5^	30	30	30	30	30
CMC ^6^	30	30	30	30	30
Total	1000	1000	1000	1000	1000
Proximate composition (%)					
Moisture	9.00	9.20	9.49	9.70	9.90
Protein	30.12	30.29	30.65	30.02	30.43
Lipid	6.23	6.42	6.21	6.31	6.31
Fibre	6.00	7.23	7.31	7.23	7.02
Ash	10.05	9.06	9.23	9.27	9.34
NFE ^7^	38.60	37.80	37.11	37.47	37.00

^1^ LFM: Local fish meal, crude protein, 39.50%; crude lipid 8.50%; ash, 18.20%; moisture, 12.00% ^2^ FWM: Fermented water spinach meal, crude protein, 30.38%; crude lipid, 3.00%; ash, 13.50%; crude fibre, 11.73%; moisture, 10.82% ^3^ CGM: Corn germ meal ^4^ DDGS: Distillery dry grain soluble ^5^ Vitamin and mineral premix (g/kg) Vitamin A, D, E, K, C, B_1_, B_2_, B_6_, B_12_, KCL, 90; KI, 0.04; CaHPO_4_·2H_2_O, 500; NaCl, 40; CuSO_4_·5H_2_O, 3; ZnSO_4_·7H_2_O, 4; CoO_4_, 0.02; FeSO_4_·7H_2_O, 20; MnSO_4_·H_2_O, 3: CaCo_3_, 215; MgOH, 124: Na_2_SeO_3_, 0.03; NaF1 ^6^ CMC: Carboxymethyl cellulose (binder) ^7^ NFE: Nitrogen-free extract.

**Table 2:** Fatty acid profiles of WM and FWM (% of total fatty acids detected).

Fatty Acids	Ingredients (%)	
	WM	FWM
4:00	1.362	4.426
6:00	0.465	0.408
8:00	12.040	12.850
10:00	2.471	3.066
12:00	0.449	0.510
13:00	4.930	0.916
14:00	0.640	0.722
15:00	0.798	1.114
16:00	19.436	21.144
17:00	0.879	1.087
18:00	5.153	6.166
20:00	2.306	3.067
21:00	1.041	3.314
22:00	2.013	3.136
24:00	1.349	2.152
14:1n5	4.549	2.198
15:1n5	9.029	4.862
16:1n7	3.446	0.714
17:1n7	1.801	2.151
18:1n9	4.565	4.104
20:1n9	1.009	3.114
24:1n9	0.362	0.461
18:2n6	5.020	0.466
20:2n6	1.201	1.756
18:3n6	1.103	3.158
20:3n6	1.234	3.546
20:4n6	1.928	3.405
20:3n3	4.189	0.552
22:6n3	1.120	1.367

Abbreviation: WM, Water spinach meal; FWM, Fermented water spinach meal.

**Table 3:** Physical properties and palatability examination of catfish diets.

Parameters			Diets (% FWM)		
	0	25	50	75	100
Feed diameter (mm)	2.01 ± 0.01 ^a^	2.01 ± 0.02 ^a^	2.01 ± 0.02 ^a^	2.01 ± 0.02 ^a^	2.01 ± 0.01 ^a^
ER (%)	2.01 ± 0.01 ^a^	2.01 ± 0.02 ^a^	2.01 ± 0.02 ^a^	2.01 ± 0.02 ^a^	2.01 ± 0.01 ^a^
Bulk density (kg/m^3^)	446.97 ± 4.33 ^a^	445.67 ± 3.06 ^a^	447.00 ± 2.00 ^a^	446.66 ± 4.04 ^a^	445.65 ± 4.01 ^a^
PDI (%)	99.65 ± 0.20 ^a^	99.56 ± 0.33 ^a^	99.60 ± 0.18 ^a^	99.49 ± 0.33 ^a^	99.58 ± 0.27 ^a^
Floatability (%)	100.00 ± 0.00 ^a^	99.67 ± 0.58 ^a^	100.00 ± 0.00 ^a^	99.67 ± 0.58 ^a^	99.67 ± 0.58 ^a^
Water stability (%)	77.76 ± 0.10 ^a^	77.68 ± 0.21 ^a^	77.82 ± 0.03 ^a^	77.76 ± 0.10 ^a^	77.76 ± 0.13 ^a^
Palatability	++++	++++	++++	++++	+++

Values are expressed as mean ± SD Abbreviation: ER, Expansion rate; PDI, Pellet Durability Index. The same superscripts alphabet in each row denotes no statistically different (*p* > 0.05); +: Broodfish took <25% of the supplemental diets within 5 minutes; ++: Broodfish consumed <50% of the supplemented diets within 5 minutes; +++: Broodfish took <75% of the supplemented diets within 5 minutes; ++++: Broodfish consumed <100% of the supplemented diets within 5 minutes.

**Table 4:** Bacterial loads (TB and LAB) estimation in diets and intestine.

Parameters			Diets (% FWM)		
	0	25	50	75	100
Feed					
TB (CFU/g) × 10^9^	1.44 ± 0.07 ^e^	4.67 ± 0.05 ^d^	7.59 ± 0.08 ^c^	11.52 ± 0.06 ^b^	15.90 ± 0.02 ^a^
LAB (CFU/g) × 10^7^	1.52 ± 0.03 ^e^	4.20 ± 0.31 ^d^	6.32 ± 0.43 ^c^	8.12 ± 0.02 ^b^	11.34 ± 0.08 ^a^
Intestine					
TB (CFU/g) × 10^9^	1.27 ± 0.05 ^d^	4.82 ± 0.03 ^c^	8.41 ± 0.38 ^a^	5.40 ± 0.10 ^b^	5.10 ± 0.10 ^b, c^
LAB (CFU/g) × 10^7^	1.63 ± 0.02 ^d^	4.41 ± 0.11 ^c^	6.15 ± 0.07 ^a^	5.98 ± 0.08 ^b^	5.85 ± 0.08 ^b^

Values are expressed as mean ± standard deviation (*n* = 3). Abbreviation: TB, Total Bacteria; LAB, Lactic Acid Bacteria. Different superscripts alphabet in each row denotes statistically different (*p* < 0.05).

**Table 5:** Water parameters of the fish holding experimental tanks.

Parameters			Diets (% FWM)		
	0	25	50	75	100
Temperature (°C)	29.27 ± 0.07 ^a^	29.32 ± 0.04 ^a^	29.25 ± 0.07 ^a^	29.26 ± 0.01 ^a^	29.29 ± 0.03 ^a^
Pressure (mm)	752.45 ± 0.04 ^a, b^	752.60 ± 0.15 ^a^	752.49 ± 0.14 ^a, b^	752.44 ± 0.07 ^a, b^	752.39 ± 0.02 ^b^
Conductivity (Siemens/meter)	335.84 ± 2.52 ^b^	361.91 ± 12.85 ^a^	348.73 ± 21.14 ^a, b^	332.61 ± 5.64 ^b^	330.41 ± 9.39 ^b^
TDS (ppm)	191.85 ± 9.43 ^a, b^	192.92 ± 4.79 ^a^	191.07 ± 10.79 ^a, b^	182.38 ± 8.08 ^a, b^	177.01 ± 3.21 ^b^
Salinity (ppt)	0.08 ± 0.01 ^a^	0.09 ± 0.01 ^a^	0.11 ± 0.04 ^a^	0.07 ± 0.01 ^a^	0.08 ± 0.00 ^a^
DO (ppm)	5.38 ± 0.08 ^a^	5.26 ± 0.03 ^a^	5.37 ± 0.11 ^a^	5.43 ± 0.08 ^a^	5.40 ± 0.13 ^a^
pH	7.10 ± 0.09 ^a^	7.07 ± 0.07 ^a^	7.10 ± 0.02 ^a^	7.14 ± 0.06 ^a^	7.10 ± 0.02 ^a^
Ammonia (ppm)	0.13 ± 0.03 ^a, b^	0.10 ± 0.03 ^b, c^	0.13 ± 0.01 ^a, b^	0.16 ± 0.03 ^a^	0.08 ± 0.00 ^c^
Nitrite (ppm)	0.10 ± 0.05 ^a^	0.06 ± 0.02 ^a^	0.10 ± 0.01 ^a^	0.12 ± 0.02 ^a^	0.07 ± 0.01 ^a^
Nitrate (ppm)	0.67 ± 0.09 ^a^	0.72 ± 0.09 ^a^	0.72 ± 0.24 ^a^	0.56 ± 0.09 ^a^	0.72 ± 0.09 ^a^

Values are expressed as mean ± standard deviation. Abbreviation: TDS, Total Dissolved Solid; DO, Dissolved Oxygen. Different superscripts alphabet in each row denotes statistical differences (*p* < 0.05).

**Table 6:** Growth and reproductive variables of broodstock fed with various levels of FWM diets for 90 days.

Parameters			Diets (% FWM)		
	0	25	50	75	100
Initial weight (g)	65.53 ± 0.63 ^a^	66.08 ± 0.35 ^a^	65.92 ± 0.44 ^a^	65.40 ± 0.66 ^a^	66.11 ± 0.38 ^a^
Final weight (g)	70.71 ± 0.31 ^c^	71.60 ± 0.64 ^c^	78.52 ± 0.59 ^b^	78.42 ± 0.17 ^b^	90.17 ± 2.14 ^a^
WG (g)	5.18 ± 0.73 ^c^	5.52 ± 0.29 ^c^	12.60 ± 0.24 ^b^	13.02 ± 0.81 ^b^	24.06 ± 2.21 ^a^
SGR (%/day)	0.08 ± 0.01 ^c^	0.09 ± 0.00 ^c^	0.20 ± 0.01 ^b^	0.20 ± 0.02 ^b^	0.35 ± 0.03 ^a^
SR (%)	93.33 ± 5.77 ^a^	100.00 ± 0.00 ^a^	100.00 ± 0.00 ^a^	100.00 ± 0.00 ^a^	96.67 ± 5.77 ^a^
Fecundity (eggs/kg) × 10^4^	11.82 ± 0.99 ^e^	17.95 ± 0.16 ^d^	24.70 ± 0.25 ^c^	29.36 ± 0.35 ^b^	33.66 ± 0.15 ^a^
GSI	9.99 ± 0.43 ^e^	13.87 ± 0.78 ^d^	15.00 ± 0.99 ^c^	16.37 ± 1.05 ^b^	17.53 ± 0.69 ^a^
HSI	0.94 ± 0.13 ^b, c^	0.78 ± 0.49 ^c^	1.3 ± 0.20 ^a^	0.94 ± 0.12 ^b, c^	1.06 ± 0.08 ^b^
VSI	1.16 ± 0.21 ^b^	1.01 ± 0.08 ^c^	1.28 ± 0.12 ^a^	1.33 ± 0.16 ^a^	1.11 ± 0.10 ^b^
IPF	0.71 ± 0.03 ^a^	0.60 ± 0.05 ^b^	0.24 ± 0.06 ^c^	0.24 ± 0.05 ^c^	0.22 ± 0.04 ^c^
FCR	2.20 ± 0.10 ^a^	2.05 ± 0.04 ^b^	1.91 ± 0.08 ^c^	1.89 ± 0.02 ^c^	1.69 ± 0.05 ^d^
FCE	0.46 ± 0.02 ^d^	0.49 ± 0.01 ^c^	0.52 ± 0.02 ^b^	0.53 ± 0.01 ^b^	0.59 ± 0.02 ^a^
PER	1.52 ± 0.07 ^d^	1.62 ± 0.04 ^c^	1.75 ± 0.08 ^b^	1.76 ± 0.02 ^b^	1.97 ± 0.06 ^a^
OD (mm)	3.00 ± 0.11 ^c^	3.11 ± 0.12 ^b^	3.25 ± 0.18 ^a^	2.66 ± 0.19 ^d^	2.99 ± 0.04 ^c^
OT (h)	13.05 ± 1.00 ^a^	12.28 ± 0.39 ^b^	10.35 ± 0.36 ^c^	10.21 ± 0.16 ^c^	10.28 ± 0.15 ^c^
Spawning response (%)	66.67 ± 17.74 ^b^	100.00 ± 0.00 ^a^	100.00 ± 0.00 ^a^	100.00 ± 0.00 ^a^	100.00 ± 0.00 ^a^
FR (%)	82.66 ± 12.31 ^c^	98.23 ± 0.23 ^b^	100.00 ± 0.00 ^a^	100.00 ± 0.00 ^a^	98.34 ± 1.56 ^b^
OC	Pink & reddish	Pink & reddish	Pink & reddish	Pink & reddish	Pink & reddish
OW (mg/egg)	0.57 ± 0.03 ^d^	0.76 ± 0.05 ^c^	1.01 ± 0.03 ^a^	0.92 ± 0.14 ^a, b^	0.89 ± 0.18 ^b^
Egg ripe (%)	78.12 ± 4.12 ^c^	89.34 ± 5.23 ^b^	98.86 ± 2.16 ^a^	92.43 ± 7.34 ^a, b^	88.67 ± 4.67 ^b^

Values are expressed as mean ± standard deviation (*n* = 3). Abbreviation: WG, Weight Gain; SGR, Specific Growth Rate; SR, Survival Rate; GSI, Gonadosomatic index; HSI, Hepatosomatic index; VSI, Visceral somatic index; IPF, Intraperitoneal fat; FCR, Feed Conversion Ratio; FCE, Feed Conversion Efficiency; PER, Protein Efficiency Ratio; OD, Ovipositor Diameter; OT, Ovulation time; FR, Fertilization Rate; OC, Ovipositor Color; OW, Oocyte Weight. Different superscripts alphabet in each row denotes statistical differences (*p* < 0.05).

**Table 7:** Hematological parameters of broodstock fed with different experimental diets for 90 days.

Parameters			Diets (% FWM)		
	0	25	50	75	100
WBC (10^3^/μL)	100.33 ± 1.15 ^c^	111.97 ± 1.05 ^b^	126.05 ± 5.37 ^a^	108.30 ± 5.47 ^b^	112.07 ± 2.52 ^b^
LYM (%)	82.14 ± 2.69 ^c^	87.36 ± 2.13 ^b^	97.00 ± 2.00 ^a^	81.00 ± 0.94 ^c^	82.47 ± 4.11 ^c^
MON (%)	10.96 ± 1.61 ^b^	12.38 ± 0.47 ^b^	15.60 ± 2.02 ^a^	12.10 ± 0.95 ^b^	11.43 ± 1.38 ^b^
EOS (%)	0.02 ± 0.01 ^b^	0.03 ± 0.01 ^a, b^	0.06 ± 0.03 ^a^	0.03 ± 0.02 ^a, b^	0.04 ± 0.01 ^a, b^
BAS (%)	0.04 ± 0.01 ^a^	0.05 ± 0.03 ^a^	0.07 ± 0.02 ^a^	0.04 ± 0.01 ^a^	0.06 ± 0.01 ^a^
GRA (10^3^/μL)	4.23 ± 0.28 ^b^	5.35 ± 0.66 ^a, b^	4.64 ± 0.54 ^b^	5.41 ± 1.43 ^a, b^	6.56 ± 0.66 ^a^
RBC (10^3^/μL)	1.93 ± 0.08 ^c^	2.34 ± 0.07 ^b^	2.75 ± 0.23 ^a^	2.21 ± 0.14 ^b^	2.17 ± 0.04 ^b^
HCT (%)	25.75 ± 1.08 ^a^	26.47 ± 0.45 ^a^	26.27 ± 2.37 ^a^	26.34 ± 0.57 ^a^	27.04 ± 0.96 ^a^
HGB (g/dL)	6.32 ± 0.98 ^c^	8.66 ± 0.66 ^b^	11.53 ± 1.55 ^a^	7.93 ± 1.05 ^b, c^	7.32 ± 0.41 ^b, c^
MCV (μm^3^)	124.00 ± 5.29 ^a, b^	122.28 ± 3.45 ^a, b^	128.33 ± 2.08 ^a^	117.12 ± 1.03 ^b^	118.72 ± 4.33 ^b^
MCH (pg)	38.00 ± 1.73 ^b^	40.41 ± 0.50 ^a, b^	40.69 ± 0.44 ^a^	39.03 ± 0.90 ^a, b^	39.56 ± 1.95 ^a, b^
MCHC (g/dL)	29.93 ± 0.90 ^a, b^	30.72 ± 0.44 ^a^	30.89 ± 0.60 ^a^	27.90 ± 1.06 ^c^	29.14 ± 0.15 ^b, c^
MPV (μm^3^)	5.64 ± 0.46 ^a, b^	5.75 ± 0.48 ^a, b^	4.78 ± 0.59 ^c^	5.97 ± 0.14 ^a^	5.17 ± 0.11 ^b, c^
RDW (%)	7.08 ± 0.08 ^a^	6.69 ± 0.49 ^a^	6.71 ± 0.52 ^a^	6.32 ± 0.69 ^a^	5.03 ± 0.05 ^b^
PLT (10^3^/μL)	39.29 ± 0.62 ^b^	42.64 ± 0.54 ^a, b^	44.99 ± 4.62 ^a, b^	47.29 ± 3.91 ^a^	45.66 ± 3.91 ^a^
PCT (%)	0.03 ± 0.01 ^a^	0.03 ± 0.02 ^a^	0.03 ± 0.03 ^a^	0.03 ± 0.02 ^a^	0.03 ± 0.01 ^a^
PDW (%)	5.99 ± 0.11 ^b^	7.46 ± 1.34 ^a, b^	8.99 ± 0.90 ^a^	7.03 ± 1.80 ^a, b^	7.89 ± 0.29 ^a, b^

Values are expressed as mean ± standard deviation (*n* = 3) Abbreviation: WBC: White blood cell; LYM: Lymphocytes; MON: Monocytes; EOS: Eosinophil; BAS: Basophil; GRA: Granulocytes; RBC: Red blood cell; HCT: Hematocrit; HGB: Hemoglobin; MCV: Mean Corpuscular Volume; MCH: Mean Corpuscular Hemoglobin; MCHC: Mean Corpuscular Hemoglobin Concentration; MPV: Mean Platelet Volume; RDW: Red Cell Distribution Width; PLT: Platelet; PCT: Procalcitonin; PDW: Platelet Distribution Width. Different superscripts alphabet in each row denotes statistical difference (*p* < 0.05).

**Table 8:** Serum biochemical indices of fish fed with different experimental diets for 90 days.

Parameters			Diets (% FWM)		
	0	25	50	75	100
GLU (mg/dL)	53.92 ± 2.10 ^d^	63.81 ± 5.45 ^c^	70.52 ± 2.78 ^b^	77.86 ± 2.03 ^a^	82.85 ± 2.57 ^a^
CREA (mg/dL)	0.11 ± 0.02 ^c^	0.15 ± 0.03 ^b, c^	0.12 ± 0.02 ^c^	0.22 ± 0.09 ^a, b^	0.30 ± 0.05 ^a^
TBIL (mg/dL)	0.10 ± 0.02 ^b^	0.13 ± 0.01 ^b^	0.12 ± 0.02 ^b^	0.18 ± 0.03 ^a^	0.21 ± 0.03 ^a^
SGPT (u/L)	30.66 ± 0.39 ^c^	41.00 ± 1.00 ^b^	51.40 ± 9.03 ^a^	42.61 ± 0.57 ^b^	45.73 ± 3.57 ^a, b^
S. Urea (mg/dL)	13.66 ± 0.67 ^c^	10.56 ± 1.58 ^d^	9.94 ± 0.03 ^d^	19.10 ± 1.48 ^b^	24.50 ± 2.62 ^a^
SGOT (u/L)	34.22 ± 1.95 ^c^	37.26 ± 0.59 ^c^	64.35 ± 4.03 ^a^	52.05 ± 7.92 ^b^	37.80 ± 6.73 ^c^
ALB (g/dL)	0.70 ± 0.05 ^d^	0.77 ± 0.02 ^d^	0.94 ± 0.05 ^c^	1.18 ± 0.04 ^b^	1.40 ± 0.10 ^a^
ALKP (u/L)	9.57 ± 0.50 ^d^	11.48 ± 1.19 ^c^	12.07 ± 0.06 ^b, c^	13.03 ± 0.08 ^a, b^	13.27 ± 0.57 ^a^
CHOL (g/dL)	10.63 ± 0.99 ^b^	8.83 ± 0.29 ^c, d^	7.80 ± 0.96 ^d^	9.70 ± 0.52 ^b, c^	12.42 ± 0.47 ^a^
TP (g/dL)	2.95 ± 0.06 ^c^	3.18 ± 0.08 ^b^	3.46 ± 0.02 ^a^	3.42 ± 0.10 ^a^	3.51 ± 0.10 ^a^
GLOB (g/dL)	2.07 ± 0.06 ^c^	2.33 ± 0.15 ^b^	2.54 ± 0.08 ^a^	2.53 ± 0.12 ^a^	2.64 ± 0.03 ^a^
ALT (u/L)	11.63 ± 1.59 ^d^	14.20 ± 2.55 ^d^	20.66 ± 1.11 ^c^	26.62 ± 2.33 ^b^	34.27 ± 2.81 ^a^
AST (u/L)	63.04 ± 2.07 ^d^	83.03 ± 3.57 ^c^	86.00 ± 7.81 ^c^	115.63 ± 4.74 ^b^	144.00 ± 4.36 ^a^
GGT (u/L)	0.89 ± 0.03 ^b^	0.92 ± 0.03 ^b^	0.89 ± 0.04 ^b^	0.96 ± 0.03^b^	1.10 ± 0.13 ^a^

Values are expressed as mean ± standard deviation (*n* = 3). Abbreviation: GLU: Glucose; CREA: Creatine; TBIL: Total Bilirubin; SGPT: Serum Glutamic Pyruvic Transaminase; SGOT: Serum Glutamic Oxaloacetic Transaminase; ALB: Albumin; ALKP: Alkaline phosphatase; CHOL: Cholesterol; TP: Total Protein; GLOB: Globulin; ALT: Alanine Aminotransferase; AST, Aspartate Aminotransferase; GGT: Gamma-glutamyl transferase. Different superscripts alphabet in each row denotes statistically different (*p* < 0.05).

## Data Availability

Not applicable.
